# A Novel Technique for the Extraction of Whiteness and Yellowness Indices of Sugar Based on Digital Color Imaging

**DOI:** 10.3390/jimaging12090448

**Published:** 2026-09-16

**Authors:** Leila Ziafar, Keivan Ansari, Masoud Honarvar, Vahid Mohammadi

**Affiliations:** 1 Department of Food Science and Technology, Faculty of Agriculture, SR.C. Islamic Azad University, Tehran 14515-775, Iran; leila.ziafar@iau.ac.ir (L.Z.); m.honarvar@srbiau.ac.ir (M.H.); 2 Department of Color Imaging and Color Image Processing, Color Physics Faculty, Institute for Color Science and Technology, Tehran 16765-654, Iran; 3 Université Bourgogne Europe, IMVIA, UR 7535, 21000 Dijon, France

**Keywords:** sugar, scanner, RGB image, color values, yellowness index

## Abstract

The visual whiteness of sugar is a key factor in determining its quality and purity. Consequently, the development of rapid and cost-effective methods for the color evaluation of sugar samples is of critical importance to the sugar industry. This study aims to introduce a method for the measurement of whiteness index (WI) and yellowness index (YI) of sugar based on a commercial digital scanner. In this regard, sugar samples were collected across three different cultivation seasons. The color images of sugar samples were obtained using a digital scanner, and the color indices were extracted based on four WI and two YI formulas. The results were then compared with the standard color measurement technique based on spectrophotometry. It was observed that the proposed technique led to a correlation coefficient of 0.947 for the estimation of WI. For both YI formulas, the correlation of 0.944 was obtained. The results indicate that the proposed technique has a remarkable performance in the estimation of sugar color indices and offers a simple, easy-to-implement and fast technique for industry.

## 1. Introduction

Sugar plays a central role in the food industry as a multifunctional ingredient that provides rapid energy, enhances taste, contributes to texture through browning and crystallization, and acts as a preservative by reducing water activity and extending shelf life [1]. Sugar is consumed globally, with a production volume of 182.2 million tons in 2023/24 [2]. White sugar, the most widely used sweetener, is sucrose—a disaccharide composed of one glucose molecule and one fructose molecule. It is typically extracted from sugarcane or sugar beets and refined into a white crystalline product [3]. Additionally, the physical and chemical properties of sugar, such as its crystalline structure and susceptibility to browning reaction, directly affect its color during processing and storage.

Whiteness and yellowness indices are critical quality indicators in the sugar industry because they directly reflect the degree of refinement, purity, and visual quality of the product [4,5]. The whiteness index measures how close a sugar sample is to an ideal white, signaling high purity, while the yellowness index quantifies the presence of color impurities like residual molasses or by-products from incomplete processing [6,7]. The color of sugar arises from various compounds, including flavonoids, phenolic substances, and pigments that interact with reducing sugars—directly influencing both the juice color and sugar quality [8]. Whiteness and yellowness indices are derived mathematically from the color coordinates (X, Y, Z) obtained through colorimetric analysis. These indices represent specific interpretations of color purity or impurity, rather than the color values themselves [9]. Thus, while XYZ values describe the overall color of the sample, whiteness and yellowness provide targeted quality metrics derived from those values.

Traditionally, the process of color measurement of sugar in the industry typically involves dissolving a sugar sample in a buffer solution at a neutral pH, measuring the dry mass by refractometry, and then assessing the absorbance of the solution photometrically at a wavelength of 420 nm [10]. These methods require manipulations of samples and are costly and time-consuming. More importantly, the traditional methods cannot be used in real-time production lines. Hence, there is growing interest to provide non-contact real-time monitoring techniques for sugar color measurement. Such techniques allow for rapid and continuous assessment without disrupting the production flow. By eliminating the need for sample preparation, dissolution, and chemical reagents, modern approaches can significantly reduce measurement time and operational costs while maintaining high accuracy. Furthermore, non-contact techniques enable the detection of color variations across the entire surface of sugar crystals, providing a more representative quality assessment than point-based laboratory methods. The integration of these technologies into online production lines would allow manufacturers to monitor whiteness and yellowness indices in real time, facilitating immediate process adjustments and ensuring consistent product quality.

One of the main and common technologies that has been used for food color measurement is computer vision [11,12,13,14]. This can be achieved by using custom-made camera and lighting systems or using digital flatbed scanners [15,16,17,18]. In both scenarios, being non-contact, inexpensive, quick and accessible have caused researchers to pay more attention to the usage of computer vision for food color measurement [19,20,21,22]. Ichsan et al. [23] investigated the estimation of ethanol content in apple, orange, and grape juices using image processing techniques. They obtained digital images of the sample, extracted the RGB values and obtained color values in different color spaces (i.e., RGB, HSV, CIELAB, CMYK, CIELuv, CIEXYZ and CIELch). It was observed that the CIELAB led to the most accurate ethanol determination. In another work, Villaseñor-Aguilar et al. [24] performed the classification of maturity in orange fruit by extracting the Citrus Color Index from RGB images. The RGB values extracted from several ROIs were then converted to CIELAB values, and a model was developed to estimate the maturity. They observed strong correlation (i.e., over 0.9) between Citrus Color Index and maturity, degree Brix, and firmness. Zia et al. [25] proposed a fast method for rice quality control based on image processing. They used a flatbed scanner for image acquisition to extract color (i.e., yellowness) as well as morphological properties of rice grains. The system achieved precision 98.8% for the classification of rice as damaged or healthy.

In previous research on sugar color, there have been two main approaches. One approach has been the use of computer vision techniques for the classification of sugar based on color [26]. In other research, computer vision has been used for the extraction of chemical properties or other quality-related factors [27,28]. In a recent study, Paz et al. [26] classified sugar samples into three groups including refined sugar for the external market, refined sugar for retail market and unrefined sugar. They extracted statistical measures from RGB images and used partial least squares discriminant analysis, achieving a classification rate of 95.7%. In another study, Paz et al. [29] used RGB image data and chemometrics for sugar color prediction. They used several regression techniques, and a prediction rate with an R2 value of 0.976 was obtained. Brabec et al. [30] used a flatbed scanner for the evaluation and measurement of flour color. This method was used to classify flour into several categories of clean flour, marginally clean, or contaminated. They transformed the RGB into CIELAB and found that the coordinate L* had a significant correlation with the values measured by a colorimeter.

The extraction of color indices of sugar samples is crucial, time-consuming and expensive, making real-time online sugar quality measurements infeasible. Consequently, developing and implementing non-contact, non-invasive measurement systems has become a critical research priority for the sugar industry to meet growing demands for automation, efficiency and quality control. In this study, a commercial digital flatbed scanner was utilized for the quick and inexpensive measurement of sugar color indices. To the best of our knowledge, this is the first research on the extraction of sugar whiteness and yellowness indices using a digital scanner. The results were also compared with the actual color indices obtained from spectroscopic measurements.

## 2. Materials and Methods

### 2.1. Sugar Samples

Sugar samples were collected (Boein-Zahra sugar factory, Qazvin, Iran) based on a completely randomized method from both spring and autumn cultivations (i.e., three sets including the first spring cultivation, the autumn cultivation, and the second spring cultivation). The aim was to collect samples of different white tones from lightest to darkest tones. The samples were kept in opaque zip bags under dry conditions in a dark environment. A total of 173 samples were used for the study. For the experiments, three repetitions were taken out of each sample, leading to a total of 519 experiments. All the experiments were performed in the institute for Color Science and Technology, Tehran, Iran.

### 2.2. Image Acquisition

For the acquisition of color images, a commercial digital color scanner (Epson, Nagano, Japan, Perfection V700 Photo) was used. Table 1 presents the main software characteristics of the scanner. Before image acquisition, the scanner was turned on for a period of 15 min for stabilization reasons. For all samples, the acquisition was performed in a certain area on the scanner. A transparent cylinder was used to hold the sugar column on the scanner.

**Figure 1:**
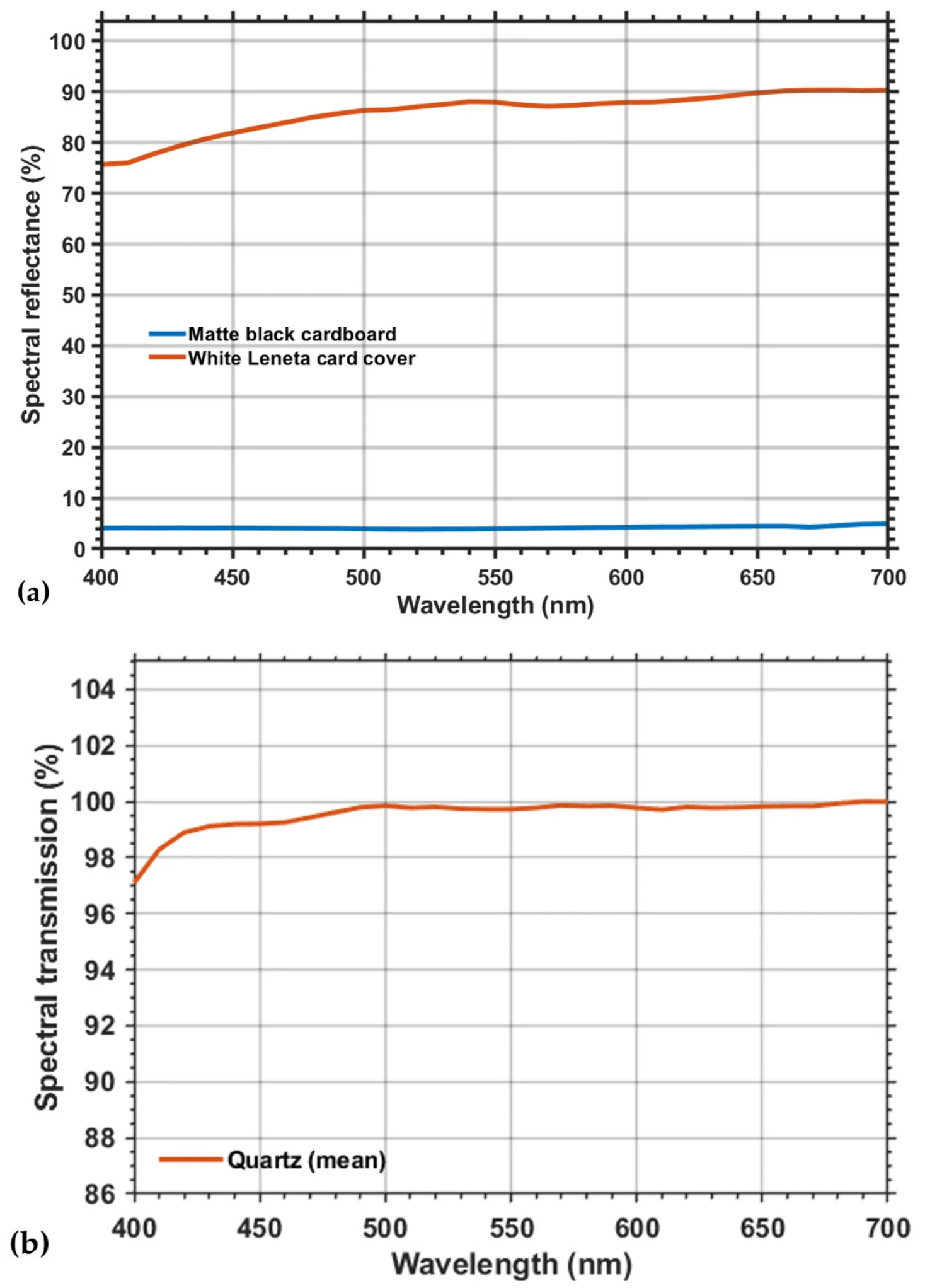
(**a**) Spectral reflectances of matte black cardboard and matte plain white Leneta card, and (**b**) spectral transmission of quartz plate.

**Figure 2:**
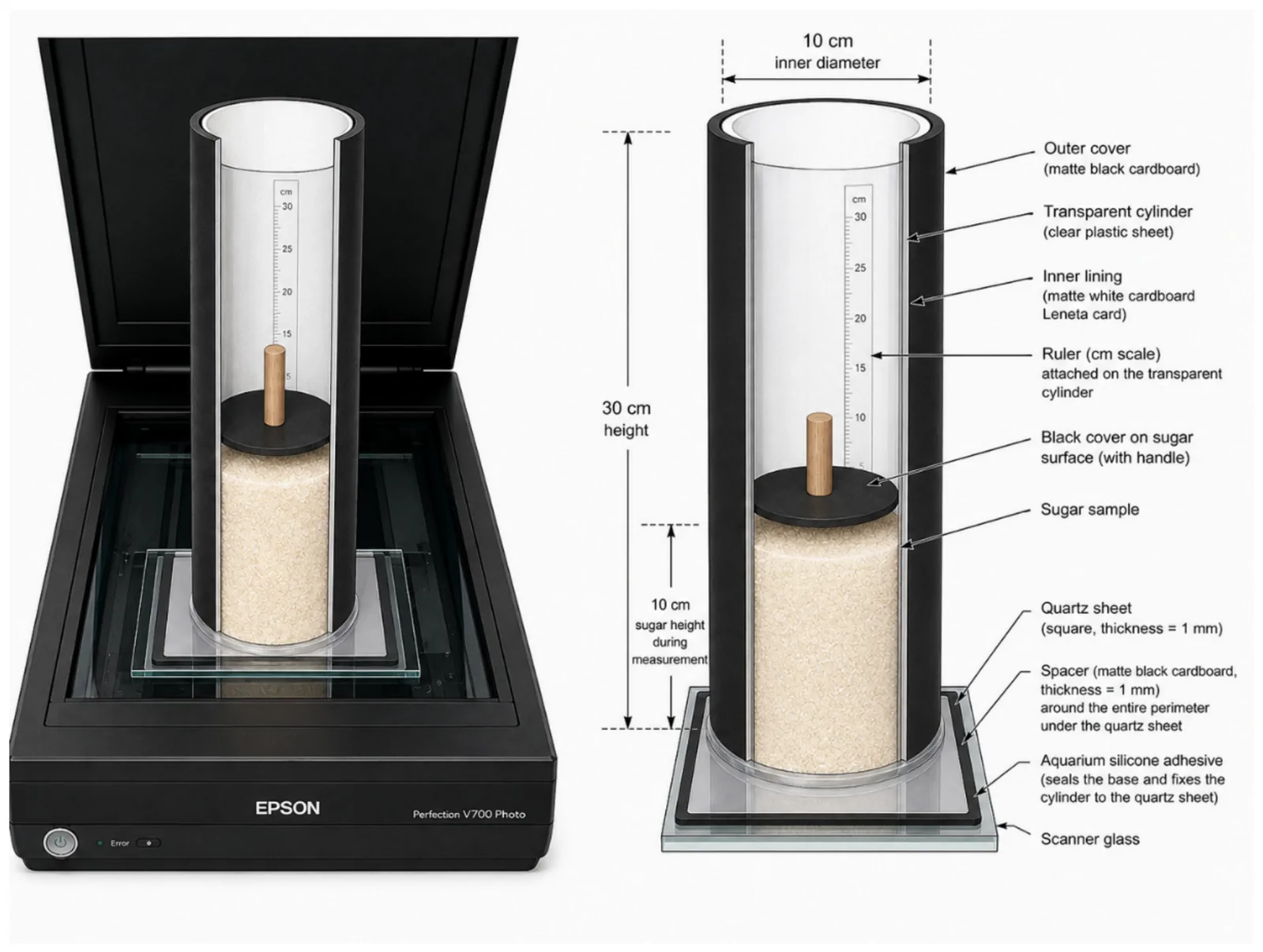
Schematic of the sample holder and the scanning setup.

A specialized sample holder was developed for image acquisition. A transparent plastic cylinder was used to hold the sugar column on the scanner. To minimize ambient-light interference, the inner surface of the cylindrical holder was covered with a matte white cardboard (Leneta card), specifically chosen for its lack of optical brightening agents. The outer surface was covered with a matte black cardboard, and the column of sugar was covered using a white Leneta card. The spectral reflectances of both covers are presented in Figure 1a. For the sugar container’s substrate, a 1 mm-thick quartz sheet was selected to minimize light effects and ensure optimal image clarity. Figure 1b illustrates the spectral transmittance of this quartz plate. The sample holder measured 10 cm in diameter and 30 cm in height. During measurements, the sugar sample maintained a height of 10 cm, ensuring sufficient thickness to prevent light transmission. Furthermore, to prevent the “rainbow phenomenon,” a 1 mm spacer was strategically placed between the scanner glass and the quartz square sheet. A schematic of this sugar sample holder and scanning setup is provided in Figure 2.

For image acquisition, the resolution was fixed at 300 dpi. The image size was then obtained as 2550 × 3509 pixels. The ICM (Internal Color Management) was turned off. The image format of TIFF and the bit-depth of 8 were set for saving output images. No resizing or image compression was applied. The gamma correction was set at 2.2, and no ICM was active. For all experiments, the same configuration was used. The complete procedure of calibration and optimization of the flatbed scanner has been presented in ref. [31], and the same protocol was strictly followed here to ensure consistency and reproducibility across all measurements.

### 2.3. Spectral Acquisition

**Figure 3:**
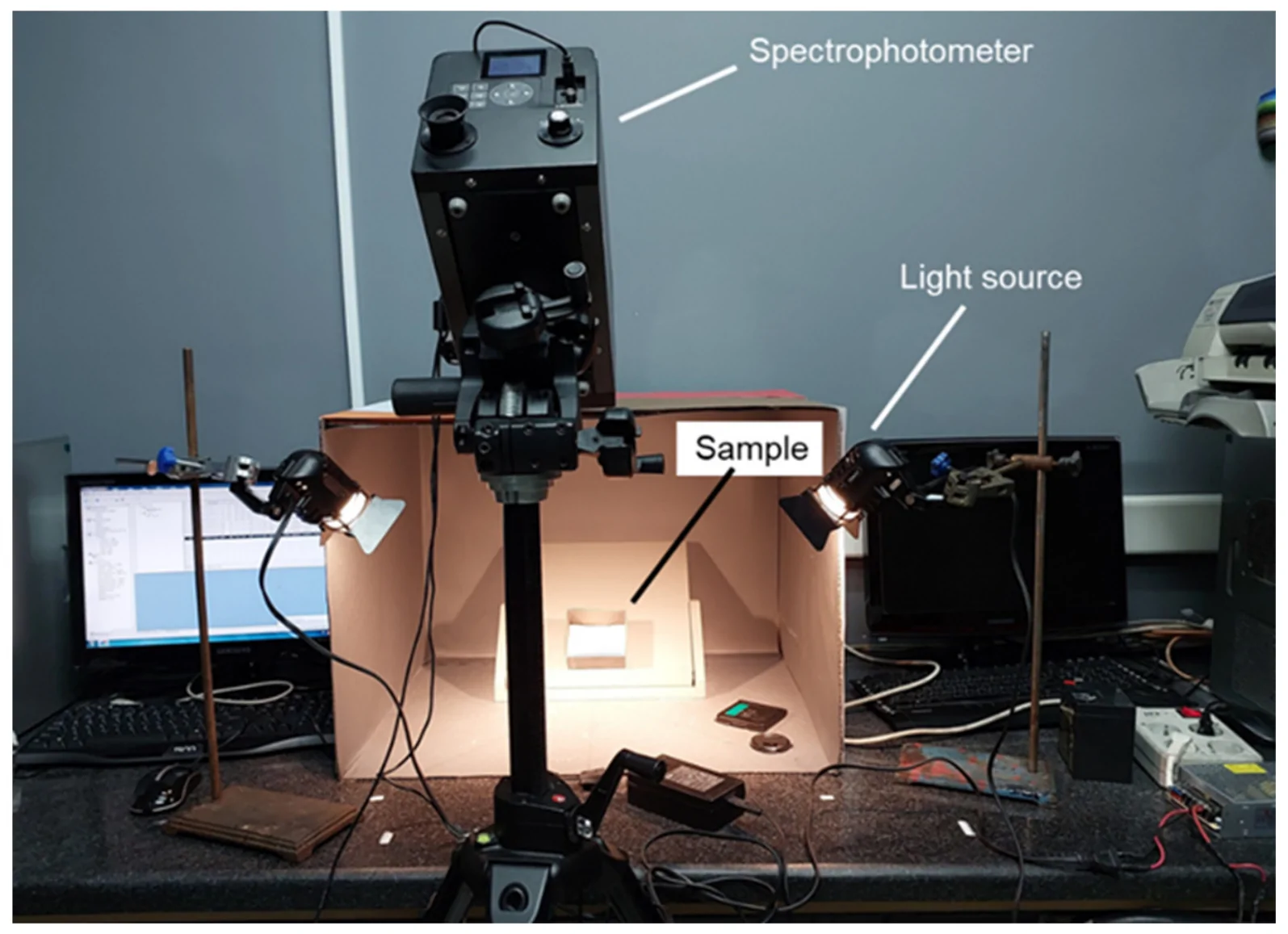
Spectral reflectance acquisition setup.

To be able to compare the obtained color values from the scanner data with actual values, the color values were measured based on the standard spectral color measurement technique using a Minolta spectrophotometer (CS-2000, Tokyo, Japan). A light room painted in average matte gray was prepared for the measurement of spectral responses of the samples (Figure 3). Two halogen lamps equipped with light diffusers were used as the light source. The angle between each light and the sample was set to 45°. A 2° standard observer spectrophotometer (CS-2000, Japan) was used for the tests. The spectrophotometer was placed at zero degrees toward the sugar sample. To do so, the sample was placed on a 45° case, and the spectrophotometer was tilted 45° downward. The XYZ color tristimulus values under standard light source D65 were then calculated using the CIE formula bellow:

(1) $$X=k\sum_{\lambda =400}^{\lambda =700}E_{\lambda }{\bar{x}}_{\lambda }P_{\lambda }\;Y=k\sum_{\lambda =400}^{\lambda =700}E_{\lambda }{\bar{y}}_{\lambda }P_{\lambda }\;Z=k\sum_{\lambda =400}^{\lambda =700}E_{\lambda }{\bar{z}}_{\lambda }P_{\lambda }$$

where $E_{\lambda }$ stands for the relative spectral power distribution of the light source; $\bar{x}$, $\bar{y}$, and $\bar{z}$ are the color matching functions of the CIE1931. $P_{\lambda }$ is the spectral reflectance obtained from the spectrophotometer measurement. In these equations, ***k*** is a normalizing factor defined as:

(2) $$k=100/\int_{\lambda =400}^{\lambda =700}E_{\lambda }{\bar{y}}_{\lambda }$$

### 2.4. Whiteness Index (WI)

Whiteness is a perceptual attribute that is described quantitatively by formulas describing its similarity to an ideal white. Several whiteness indices have been developed over the decades, each with different mathematical formulations and underlying assumptions about whiteness perception. In this study, four widely recognized WI formulas were applied to evaluate the whiteness of sugar samples from their CIEXYZ tristimulus values obtained under D65 illumination and the CIE 1931 2° standard observer.

#### 2.4.1. Berger Whiteness Index

The Berger formula is one of the oldest WI formulas, which was quite popular for decades. The Berger formula considers all three CIE tristimulus values (*X*, *Y*, *Z*). In this formula, while *Z* (blue stimulation) contributes positively, *X* (red stimulation) contributes negatively to the whiteness score. This reflects the perceptual principle that white surfaces with a slight blue undertone appear “whiter” than those with a yellowish or reddish cast. The formula for two-degree observer is as follows [32]:

*WI_Berger_* = *Y* + 3.452*Z* − 3.908*X* (3)

where *X*, *Y* and *Z* are the CIE*XYZ* color values.

#### 2.4.2. Taube Whiteness Index

Unlike the Berger formula, which uses all three tristimulus values, the Taube whiteness index simplifies the calculation by considering only two coordinates: *Y* (luminance) and *Z* (blue stimulation). This simplification is based on the observation that the dominant perceptual factor distinguishing white from near-white is the balance between lightness and blue content. The formula is particularly sensitive to blue tint—a desirable quality in white products, as human vision tends to prefer whites with a slight blue shift over yellowish whites.

The Taube formula is defined for the CIE 1931 2° standard observer as:

*WI_Taube_* = 3.727*Z* − 3*Y* (4)

#### 2.4.3. Hunter Whiteness Index

The Hunter whiteness index introduces an important modification by normalizing the whiteness value by the square root of luminance (*Y*). This normalization accounts for the fact that the human eye’s ability to perceive color differences varies with brightness—at higher lightness levels, even small chromatic deviations become more noticeable. The formula uses both the *Z* (blue) and *X* (red) coordinates, with *Z* contributing positively and *X* negatively, while *Y* appears only in the denominator as a normalization factor.

The Hunter WI for the CIE 1931 2° standard observer is calculated as:

(5) $${\mathrm{WI}}_{\mathrm{Hunter}}=\;\frac{19.297Z-11X}{\sqrt{Y}}$$

#### 2.4.4. Stensby Whiteness Index

The Stensby whiteness index is closely related to the Hunter index but differs in one critical aspect: it includes all three tristimulus values with non-zero coefficients, and its weighting scheme reveals a deliberate preference for red-shaded whites. While most whiteness formulas penalize redness (negative *X* coefficient), the Stensby formula gives *X* a positive coefficient, meaning that samples with higher red reflectance can achieve higher whiteness scores—provided this is offset by a sufficiently high *Y* (lightness) value. This characteristic made the Stensby index popular in the United States textile and paper industries, where a slightly warm (red-tinted) white was sometimes preferred over a cold (blue-tinted) white for certain applications. The formula for the CIE 1931 2° standard observer is:

(6) $${\mathrm{WI}}_{\mathrm{Stensby}}=\;\frac{19.297Z+55.251X-63.5Y}{\sqrt{Y}}$$

### 2.5. Yellowness Index (YI)

While whiteness indices measure how close a sample is to an ideal white, yellowness indices specifically quantify the degree of yellowing—a common degradation phenomenon in sugar and other food products caused by thermal processing, chemical reactions (Maillard browning) or prolonged storage. A higher yellowness index indicates a more pronounced yellow discoloration, which is generally undesirable in white sugar. In this study, two yellowness indices were applied.

#### 2.5.1. E313 Yellowness Index

The E313 yellowness index, standardized by the American Society for Testing and Materials (ASTM), is one of the most widely used measures for quantifying yellowness in near-white or off-white materials [33]. It is recommended for materials ranging from white to yellow in appearance, making it well-suited for sugar color evaluation. The index is based on the principle that yellowness corresponds to a deficiency in blue reflectance relative to red reflectance, normalized by the overall lightness of the sample. The formula is defined as:

(7) $${YI}_{E313}=100\frac{\left(C_{X}X-C_{Y}Z\right)}{Y}$$

where *C_X_* of 1.2985 and *C_Y_* of 1.1335 is used for YI calculation. In this equation, *X*, *Y* and *Z* are the CIEXYZ color values.

#### 2.5.2. BASF Yellowness Index

The BASF yellowness index, developed by the chemical company BASF, offers a simpler alternative to the ASTM E313 formula. It was specifically designed to detect yellowness arising from thermal or chemical degradation in white products—a context directly relevant to sugar processing, where heat during refining or drying can induce color changes [34]. The index has been applied across diverse industries including food, paper, dyes, plastics, and textiles, which speaks to its general utility. The formula is defined as:

(8) $${YI}_{BASF}=\frac{Z-X}{Y}$$

where *X*, *Y* and *Z* values of the color tristimulus values are calculated from the spectral reflectance of the sample under D65 illumination and a 2º standard observer (CIE 1931).

### 2.6. Repeatability Error

The repeatability error was calculated to assess the reproducibility and stability of WI and YI measurements. It was obtained by computing the deviation between the scanner-obtained color index and the actual color index, where each individual error term was defined as

Error = scanner-obtained color index − actual color index(9)

To quantify the overall repeatability, the root mean square of these errors was normalized by the mean of the actual color index values, following the formula:

(10) $$Repeatability\;Error=\frac{\sqrt{\sum {(Error}^{2})/N}}{Mean}\times 100$$

This metric provides a dimensionless percentage measure of the stability of the scanner-based measurements, with lower values indicating more consistent and reproducible WI estimation across repeated measurements.

### 2.7. Tint Analysis

While whiteness and yellowness indices provide scalar measures of overall color quality, they do not fully capture the directional nature of chromatic bias—i.e., whether a sample deviates toward blue, green, red, or purple. To address this, the Ganz tint formula was employed to evaluate the tint of the white sugar samples [35]. The Ganz formula expresses tint as a linear combination of the CIE chromaticity coordinates (x, y):

*T_Ganz_* = *mx* + *ny* + *k* (11)

where *x* and *y* are the CIE 1931 or 1964 chromaticity coordinates of the sample. In this equation, *m*, *n* and *k* are empirical coefficients. For the 2° observer, the standard Ganz formula coefficients are *m* = 931.576 and *n* = −833.467. The formula outputs a tint value; if the result is greater than zero, then the sample tends to be bluish/greenish; if it is less than zero, then the sample tends to be reddish/purple. Additionally, if the *T* is equal to zero, then the sample slightly tends toward blue and then is considered neutral. The Ganz formula is particularly valuable because it provides directional information that complements the magnitude-oriented WI and YI. For example, two sugar samples might have identical WI values but opposite tint signs.

### 2.8. Image Processing

The images of sugar samples were first cut by keeping the sugar circle and removing all external area around it. Then, the RGB values (i.e., red, green, and blue) were measured by averaging all the pixels in the circle. Then, for calculation of whiteness and yellowness indices, these RGB values were transformed to CIEXYZ values. It is noted that these RGB values were under the Gamma value of 2.2 with no ICM. As demonstrated in our previous study, where several RGB-to-XYZ conversion matrices were evaluated, this specific configuration produced the lowest color difference relative to the reference data [31]. For this conversion, therefore, the following matrix was used, which is under D65 and 2° standard observer [36]:

$$\left[\begin{array}{ccc} 0.4124564 & 0.3575761 & 0.1804375\\ 0.2126729 & 0.7151522 & 0.0721750\\ 0.0193339 & 0.1191920 & 0.9503041 \end{array}\right]$$

As the sugar samples are poured into a cylinder with a quartz sheet at the bottom, the light emitted by the scanner must pass first through the quartz sheet, then interact with the sugar crystals, and finally pass again through the quartz before reaching the scanner sensor. This double transmission through quartz reduces the amount of reflected energy captured by the scanner. As a result, a systematic and nearly constant offset appears between the XYZ values measured by the scanner and those obtained directly by the spectrophotometer, which does not involve this additional optical path. To quantify this offset, five repeated experiments were performed using a reference sugar sample, and the average difference was calculated and used as the additive bias correction. Therefore, the bias values are the average differences between the scanner-obtained XYZ and the actual XYZ values (Table 2). These bias values are added to the scanner XYZ values for all the experiments. Then, for each experiment, the corrected XYZ values are obtained as follows:

(12) $$X_{C}=B_{X}+X_{R}\;Y_{C}=B_{Y}+Y_{R}\;Z_{C}=B_{Z}+Z_{R}$$

where *B* is the bias and XYZR stands for the raw values of XYZ. All the calculations and image processing were performed using MATLAB software (2019b, Mathwroks, Natick, MA, USA).

## 3. Results

**Figure 4:**
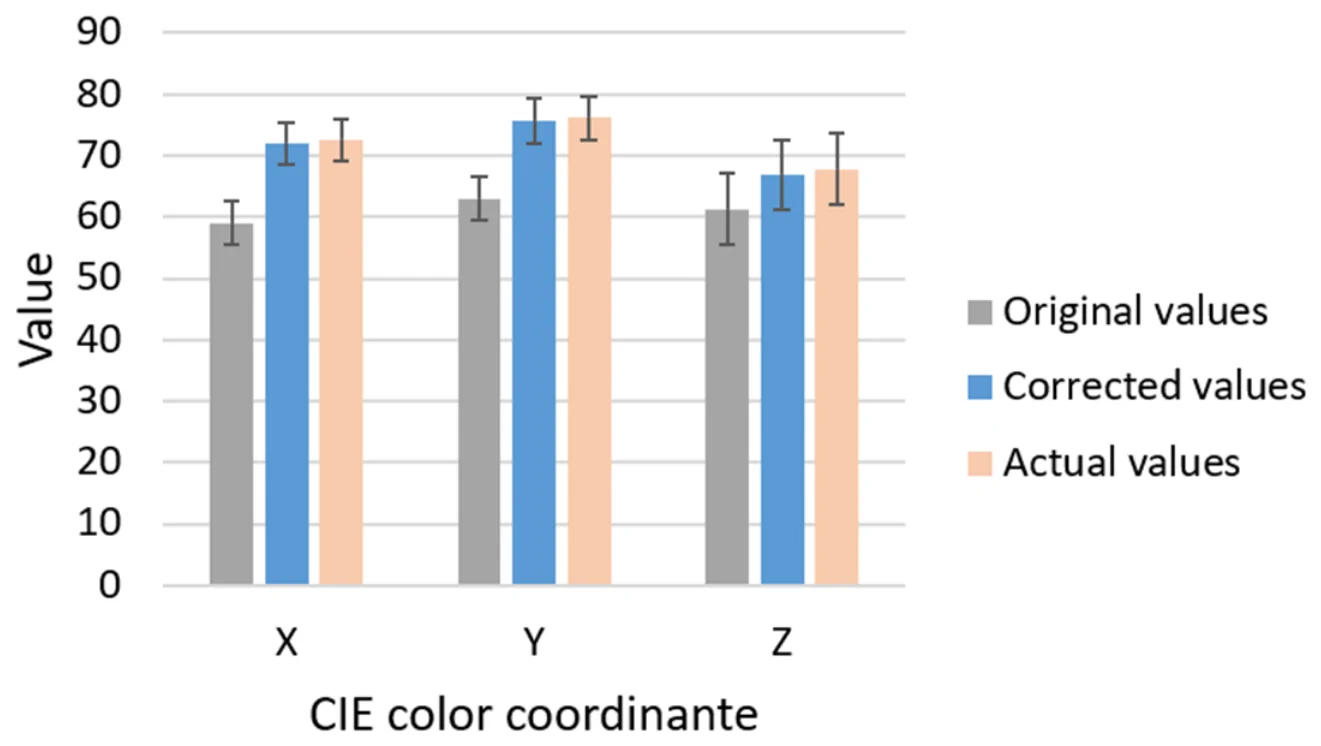
Comparison of the XYZ values obtained from scanner images and the actual values obtained by spectrophotometer.

In this work, the usage of a digital scanner was studied for the measurement of whiteness and yellowness indices of sugar samples. For this purpose, the sugar samples were scanned by the digital scanner, and the RGB values were obtained. Then, the RGB values were transformed to XYZ values, and these values were utilized for the calculation of WI and YI. The actual XYZ values of the samples were measured using the spectrophotometry technique. Figure 4 presents the average XYZ values obtained from the scanner (i.e., the raw values and the corrected values by adding the bias) and the spectrophotometer. As the figure shows, the corrected XYZ values are remarkably close to the actual values. The standard deviation error bars in the figure also confirm that variations in both measurements are quite close.

### 3.1. Whiteness Indices

**Figure 5:**
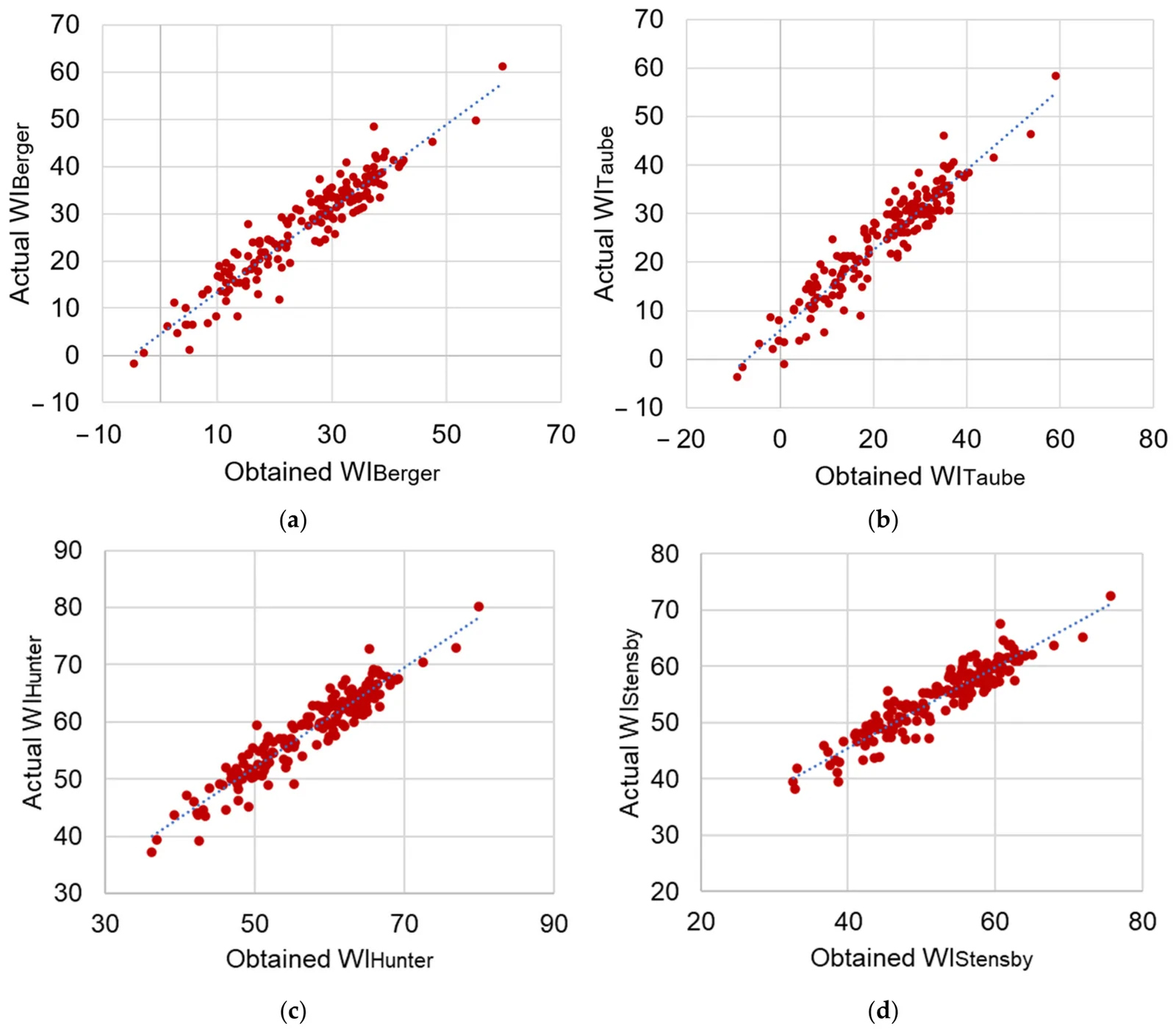
The obtained WI values from scanner images and the actual (obtained by spectrophotometry) values based on four different WI relations; (**a**) Burger, (**b**) Taube, (**c**) Hunter and (**d**) Stensby.

Four different WI measures were employed. Figure 5 depicts the relationship between the obtained WI values from the scanner and the actual values obtained by the spectrophotometer. As the figure shows, the scanner WI values are remarkably close to the actual values. The highest correlation was obtained for WI_Berger_, equivalent to 0.947, and the smallest correlation amounted to 0.932 for WI_Stensby_. Therefore, in all four WI measures, the correlation coefficient was remarkable.

The WI is a very critical measure in industry for white food products. There has been other research reported on the measurement of WI based on digital images. In an early work, Liu and Paulsen [37] proposed the measurement of corn whiteness using digital RGB images. The RGB values were transformed to the YCrCb color space, and then they defined a whiteness measure based on its coordinates. The study showed that the proposed method could discriminate corn colors just as well as humans. Quevedo et al. [38] studied the determination of bloom chocolate using WI, L* lightness and fractal Fourier method. Bloom chocolate causes a change in color and loss of gloss in the surface of chocolate, producing a grayish white appearance. RGB images were obtained and converted to L*a*b* color space. The authors found that WI and mean L* behaved similarly, while the fractal method detected the development of bloom faster. Zhao et al. [39] benefited from RGB imaging for the evaluation of mushrooms whiteness. They transformed the RGB values to CIEXYZ values and then used the Ganz whiteness. High correlation values were obtained, and the proposed technique could quantitatively evaluate the freshness of the mushrooms. Minz and Saini [40] assessed the color of flavored milk using RGB imaging. The milk samples were placed in a glass beaker of 100 mL with a diameter of 50 mm. They obtained the RGB values and transformed them into CIELAB values. The results were compared with the CIELAB values measured by a spectrophotometer. They reported that the total color difference was in the range of 0.20–6.42. Pathak et al. [41] studied the estimation of unripe banana slices using computer vision. They calculated browning index, whiteness index and color change from a computer vision system and compared the image-analysis results with a HunterLab colorimeter. They concluded that computer vision could be used for industrial-scale quality assessment.

In addition to correlation analysis, several error-based performance metrics were computed to further evaluate the predictive behavior of the scanner-derived whiteness indices (Table 3). The results show that WI_Hunter_ and WI_Stensby_ exhibit the lowest error magnitudes across all metrics, with coefficient of variation values of 12.345 and 10.999, respectively, and repeatability errors below 7.0. These relatively small deviations indicate that the scanner produces stable and consistent measurements for these two formulations. In contrast, WI_Berger_ and WI_Taube_ display higher coefficients of variation and repeatability errors, reflecting greater sensitivity to sample or imaging variability. Nevertheless, their RMSA and MAE values remain within acceptable ranges for practical industrial use. Overall, the error analysis confirms that the proposed scanner-based method achieves reliable performance, with WI_Hunter_ and WI_Stensby_ showing the strongest stability among the four whiteness formulations.

### 3.2. Yellowness Indices

**Figure 6:**
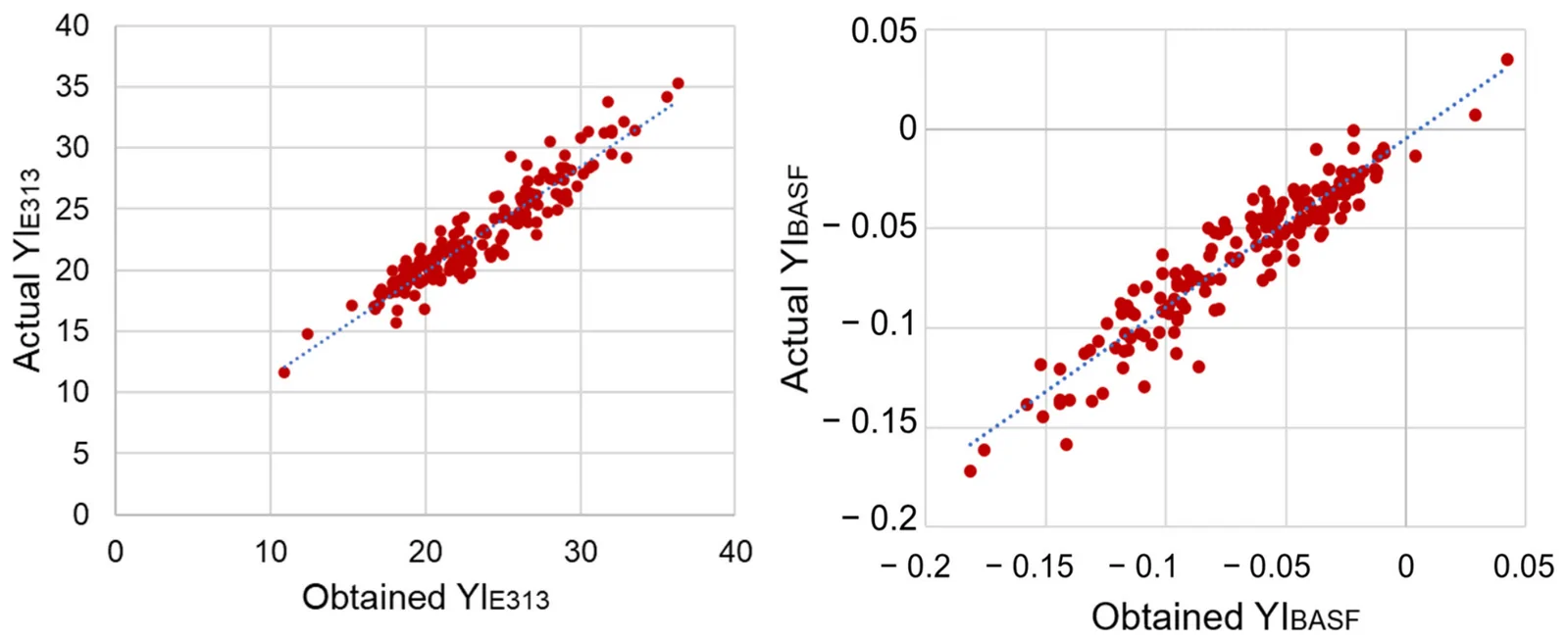
Comparison of the YI values obtained from scanner images and the actual values.

In this study, two formulas were employed for the calculation of YI. The correlation coefficient between the obtained YI values and the actual values for both measures amounted to 0.944. This shows a remarkable precision for the obtained YI values using the digital scanner. Figure 6 illustrates the relationship between the obtained and actual YI values. It is observed that the YI values obtained from the proposed method are in close relation with the actual values.

As the results show, the measurement of color indices of sugar using a digital scanner can be promising for industrial applications that require a fast and inexpensive tool for monitoring the production line. Paz et al. [29] used RGB images for the classification of sugar samples into three groups, which led to the accuracy of 95.7%. In some studies, researchers have used color data for the physio-chemical analysis of sugar properties. Alves et al. [42] imaged sugar samples using smartphone and used kNN as the regression technique for the prediction of sucrose, minerals, and color in brown sugar. The best result, the coefficient of determination of R2 equal to 92.33%, was achieved for Ca, ICUMSA color, Fe and Zn. Yeo and Saptoro [43] tried to obtained YI from RGB, XYZ and CIELAB color values by using different regression techniques. It was observed that the best result was achieved by least squares support vector regression, which outperformed principal component regression (PCR) and partial least square regression (PLSR) methods. Wasnik et al. [44] worked on the detection of adulteration in cow ghee (which is a clarified butter fat). In this study, the image acquisition was performed using a flatbed scanner, and yellowness index and whiteness index were obtained along with several other factors. It was observed that the pure ghee represented higher yellowness index and less whiteness index than that of adulterated ghee.

**Figure 7:**
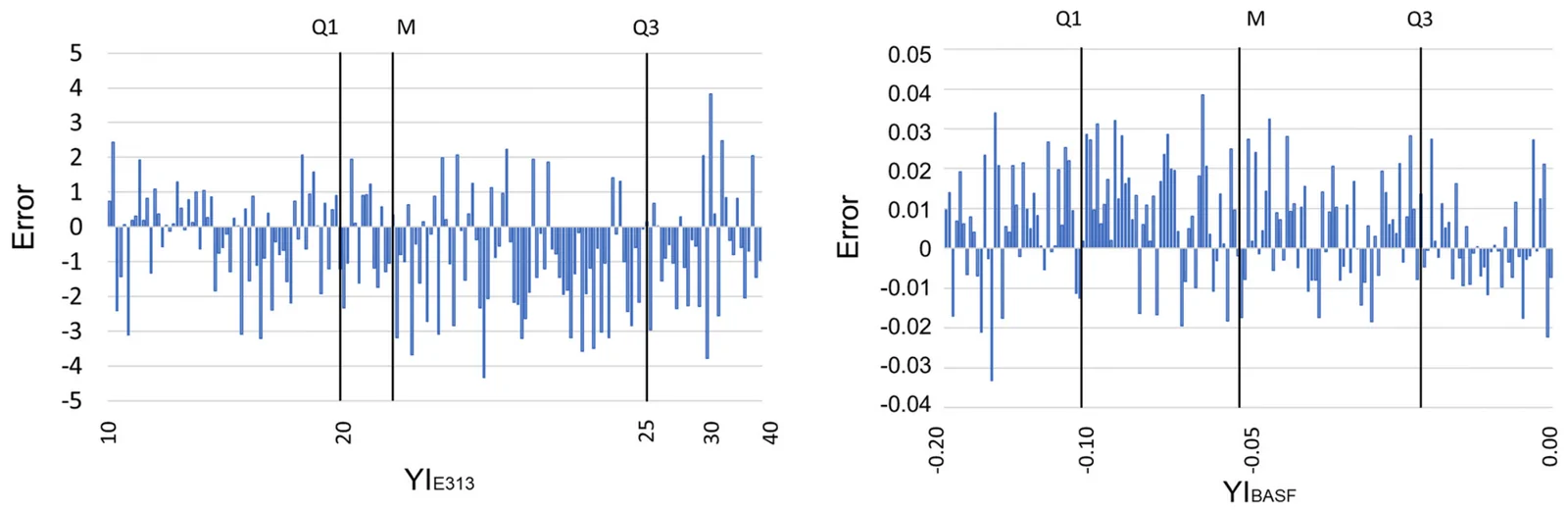
Error diagram of YI based on different quartiles.

Figure 7 represents the error between the estimated and the actual YI values. This figure helps to spot how positive and negative estimations have been performed. The quartile bars on the figure show the distribution of data. As the figure shows, 25% of the data have YIE313 of less than 19.8, and for 50% of the data, the YIE313 lies between 19.8 and 25.7. For YIBASF, the first 25% of data are less than −0.087, and 50% of data are between −0.087 and −0.036.

Beyond correlation analysis, several error-based metrics were calculated to further assess the predictive behavior of the scanner-derived yellowness indices (Table 4). The results indicate that YI-E313 exhibits relatively low error magnitudes, with an RMSA of 1.627, MAE of 1.302 and a repeatability error below 7.0, confirming that this formulation yields stable and consistent scanner-based estimates. In contrast, YI-BASF shows a substantially higher coefficient of variation and repeatability error, reflecting greater sensitivity to sample variability or imaging noise. However, its RMSA and MAE values remain very small, indicating that the absolute deviations from spectrophotometric measurements are limited despite the higher variability. Overall, the error analysis supports the conclusion that the proposed scanner method provides reliable YI predictions, with YI-E313 demonstrating the strongest stability among the two yellowness formulations.

### 3.3. Tint Analysis

**Figure 8:**
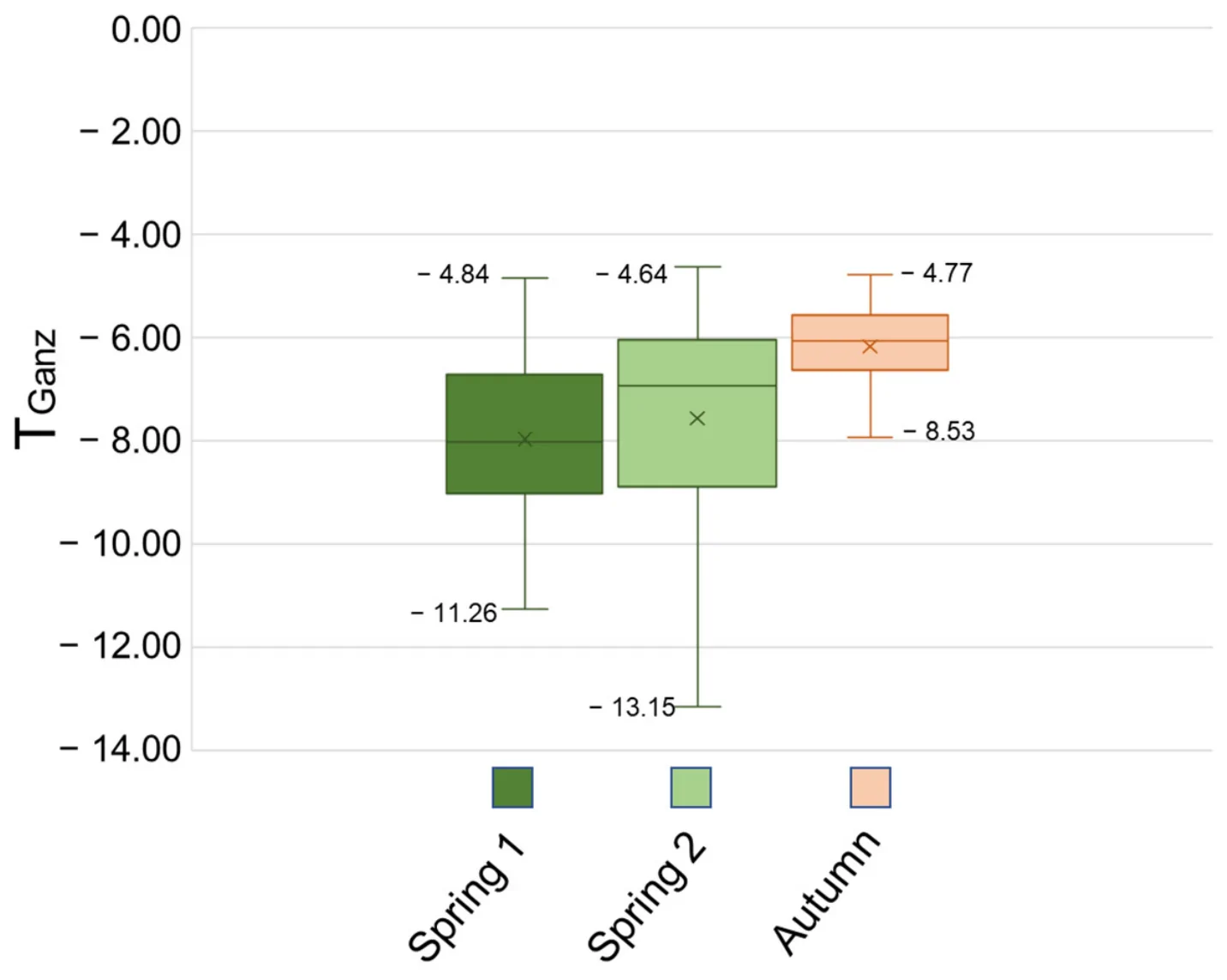
Statistical analysis of tint of sugar samples based on actual color values.

The Ganz tint parameter (T) serves as an indicator of hue deviation from a neutral reference, with negative values typically associated with a shift toward yellow-brown tones, and positive values indicating a shift toward reddish or bluish hues. As illustrated in Figure 8, all cultivation batches yielded negative T values, confirming a consistent tendency of the samples toward yellow-brown coloration. This result aligns with typical sugar color behavior and suggests the presence of non-removed colorants, such as melanins, melanoidins and caramels, which are common in raw and minimally refined sugars. While the maximum T values observed across all cultivations were similar and indicative of predominantly yellow sugar samples, the Spring-harvested samples exhibited notably greater variability. Specifically, their minimum T values reached levels associated with considerably browner hues, suggesting that these samples were more susceptible to darker coloration, possibly due to higher impurity content.

In contrast, the Autumn samples displayed a narrower range of T values, indicating more consistent color quality with limited variation. The minimum values in this group remained relatively close to the maximum, suggesting that Autumn-harvested sugars maintained a more uniform yellowish tint. This reduced variability may be attributed to more stable climatic conditions at harvest, lower levels of color-precursor compounds, or more consistent processing parameters during the Autumn season. Overall, these findings point to a seasonal influence on sugar tint, with Autumn cultivation yielding more visually uniform products.

## 4. Conclusions

This study aimed at the usage of a commercial digital scanner for the measurement of whiteness and yellowness indices of sugar samples for industry. The sugar samples in three consequent cultivation seasons were collected, and the color values were measured using a digital scanner and a spectrophotometer. The comparison of four WI equations revealed that the highest correlation between the scanner and the actual WI values were obtained for the Berger formula, equivalent to 0.947. However, for YI, formulas of both E313 and Basf led to the correlation value of 0.944 between the scanner and the actual YI values. The result of the tint analysis revealed that no sugar samples had pure white tint, and all the samples were yellowish or brownish whites. Therefore, it is observed that the use of digital scanners can provide a quick and inexpensive method for the determination of WI and YI in industrial applications. However, the bias-correction coefficients established here should be interpreted as device-specific. Extending the method to multiple scanner models remains an important direction for future work, as cross-scanner validation will be necessary to determine the generalizability of the approach and to support the development of a unified calibration strategy for industrial applications. Finally, the proposed approach focuses on establishing a controlled, scanner-based baseline method; extending the comparison to other imaging devices, such as smartphones or industrial cameras, remains an important direction for future research.

## Figures and Tables

**Table 1:** Characteristics of the digital scanner.

Scan resolution	6400 DPI (ppp) (horizontal × vertical)
Scanning area	216 mm × 297 mm (horizontal × vertical)
Color depth	Input: 24 Bit Color, Output: 24, 48 Bit Color
Optical Sensor	Matrix CCD
Light source	Cold cathode fluorescent lamp
Output resolution	50~6400 (1 dpi step), 9600, 12,800 DPI (ppp)
Reflective scanning	Monochrome: 11 s per page Color: 14 s per page measured with Format: A4, Resolution 300 dpi Color: 25 s per page measured with Format: A4, Resolution 600 dpi

**Table 2:** Calculation of the bias between the XYZ values obtained from scanner RGB values and the actual XYZ values obtained by spectrophotometer.

	Average *X*	Average *Y*	Average *Z*
Color values from RGB	60.55 ± 1.59	64.61 ± 1.67	65.39 ± 2.25
Actual value	73.44 ± 2.88	77.29 ± 3.01	70.98 ± 2.97
Bias	12.88	12.68	5.59

**Table 3:** Metrics for prediction errors of the proposed method for Whiteness indices.

	Coefficient of Variation	RMSA	MAE	Repeatability Error
WI_Berger_	39.021	3.945	3.156	15.410
WI_Taube_	43.352	4.565	3.702	20.419
WI_Hunter_	12.345	2.796	2.250	4.875
WI_Stensby_	10.999	3.682	2.946	6.990

**Table 4:** Metrics for prediction errors of the proposed method for the yellowness indices.

	Coefficient of Variation	RMSA	MAE	Repeatability Error
YI_E313_	18.260	1.627	1.302	6.975
YI_BASF_	58.540	0.015	0.012	21.510

## Data Availability

The raw data supporting the conclusions of this article will be made available by the authors on request.
